# A Bequest to the Middlesex Hospital

**Published:** 1888-03-17

**Authors:** F. Clare Melhado

**Affiliations:** Secretary-Superintendent


### A BEQUEST TO THE MIDDLESEX HOSPITAL.

IT may be of interest to your readers to hear that the executors of the late Mr. Henry Quinn have apportioned the sum of £1,200 out of his magnificent bequest to the London charities to the Middlesex Hospital.

F. CLARE MELHADO, Secretary-Superintendent.

[Can any one send us a complete list?—ED. *T. H.*]

